# Supplementary material for: BBDetector: Intelligent border binary detection in IoT device firmware based on a multidimensional feature model
Source: PLoS One. 2025 Aug 7;20(8):e0329469. doi: 10.1371/journal.pone.0329469 (PMC12331098; doi:10.1371/journal.pone.0329469)
Supplement: Attachment — Submitted filename: Response to Reviewers.docx [file pone.0329469.s001.docx]

Response to Reviewers

March 11, 2025

Guimin Zhang

Email: zh.guimin@163.com

*PLOS ONE*

Manuscript Number: [PONS-D-24-50757]

BBDetector: Intelligent border binary detection in IoT device firmware based on a multidimensional feature model

Dear Reviewers,

Thank you for your time and effort in reviewing our work and constructive suggestions on our manuscript. Based on your comments, we have carefully revised the manuscript to address all the issues raised. All revisions have been highlighted in yellow in a separate file labeled ‘Revised Manuscript with Track Changes’. Below, we provide a point-by-point response to your comments and describe the changes made to the manuscript.

**Reviewer #1**

This paper introduces a border detection scheme based on a multidimensional feature model, presenting several interesting results. Overall, the paper is well-organized and well-written.

**Comment 1**: The motivation behind this study needs to be further clarified. Specifically, the definition and importance of the border binary should be elaborated.

**Response**: We sincerely appreciate your valuable feedback on the definition and importance of border binaries. Upon reflection, we recognize that our initial explanation of border binaries was insufficient due to oversight, which may have hindered a clear understanding of their significance and the motivation behind this study. To address this, we have revised the manuscript to provide a more detailed explanation of the definition and importance of border binaries, as well as the motivation for this research. The relevant revisions can be found on Pages 5-6, Lines 202–215.

**Revision text**:

Border binaries are the executable binaries within IoT firmware that directly interact with external network data. They are responsible for receiving, parsing, and processing network requests from external sources, then passing the results to other modules within the firmware for further processing. As critical components of firmware, the importance of border binaries is reflected in two key aspects. First, they are involved not only in fundamental network communication operations, such as receiving and sending data, but also in handling critical tasks like identity authentication and firmware updates, playing a vital role in the functionality of the firmware. Second, due to the widespread vulnerabilities inherent in border binaries, they have become the primary entry point for most malicious data aiming to infiltrate the firmware, posing a significant threat to firmware security. Therefore, accurately identifying border binaries is crucial for ensuring the security of IoT devices, as the vulnerabilities in these binaries could allow attackers to breach the device’s defenses, infiltrate the firmware, and severely compromise the security of the entire IoT system.

**Comment 2**: While the authors identify three challenges in detecting border binaries, they do not adequately explain why existing state-of-the-art solutions fail to overcome these challenges.

**Response**: We thank the reviewer for raising this issue. Based on the reviewer’s suggestion, we have added a detailed explanation in the manuscript of why existing solutions fail to overcome these challenges. The relevant revisions can be found on Pages 2-3, Lines 44–68.

**Revision text**:

**C1: Selection of Border Binary Features**. Different IoT devices may have different border binaries, which vary depending on the network protocol used. Existing solutions, such as Karonte [12] and SaTC [13], exhibit notable limitations in feature selection: Karonte [12] utilizes only five features for detecting border binaries, while SaTC [13] relies on shared keywords between the web front-end and back-end. These methods do not fully capture the essential features of the border binaries, leading to inaccurate detection results. To date, there is no clear standard to guide feature selection and extraction for border binaries. Therefore, determining which features can accurately and comprehensively characterize the border binaries is the primary challenge in achieving accurate detection.

**C2: Construction of a Border Binary Dataset**. Constructing a high-quality border binary dataset is crucial for improving the robustness of border binary detection models. Existing solutions [14-16] either rely on heuristic methods to identify border binaries without using datasets, or rely on small-scale datasets, failing to allocate sufficient resources to build widely used border binary sets. Given the current lack of open-source border binary datasets for training machine learning (ML) models, it is necessary to construct such a dataset through automated tools and manual review. However, this part of the work heavily relies on expert experience and requires significant human resources.

**C3:** **Choice of a Border Binary Detection Model**. Existing solutions [9,12,17] perform poorly in identifying diverse border binaries. For example, rule-based methods [12,17] struggle to adapt to the protocols of diversified devices, while traditional machine learning-based solutions [16] exhibit limited generalization ability when encountering unseen border binaries. Therefore, selecting an appropriate border binary detection model is crucial for achieving higher accuracy and improving the efficiency of border binary detection.

**Comment 3**: Considering that the detection model training consumes considerable energy, it is important to discuss the energy consumption of nodes within IoT networks. Are these nodes active, such as LoRa nodes as discussed in “Impact of LoRa Imperfect Orthogonality: Analysis of Link-Level Performance”, or passive, like RFID nodes in “Revisiting RFID Missing Tag Identification: Theoretical Foundation and Algorithm Design”? Clarifying this critical concern and referencing these studies would enhance the paper’s practicality and relevance.

**Response**: We sincerely thank the reviewer for their valuable suggestions. Inspired by the recommended literature, we have supplemented the related work with a method for locating border binaries through dynamic emulation of protocol services that receive external network inputs in firmware, categorizing it as the third category of border binary detection method. However, given the high computational resource requirements of dynamic emulation and the inherent challenges of firmware emulation, this paper primarily focuses on static heuristic-based methods rather than dynamic emulation approaches. Although training the detection model consumes some energy, our model has lower energy consumption during the detection phase and avoids the additional resource demands of dynamic emulation.

Furthermore, our current detection model mainly targets the code processing logic in the static environment of firmware and does not specifically distinguish between active and passive characteristics of nodes. While dynamic emulation methods may uncover more hidden border binaries, their implementation complexity and resource consumption are significant, and thus, we have listed them as a direction for future research. We have referenced the studies mentioned by the reviewer (references [26] and [28]) in the relevant section to enhance the practicality and relevance of our work. The specific revisions are on Page 4, Lines 116-120.

**Revision text**:

An analysis of existing research shows that methods for identifying critical binaries in IoT firmware can be divided into three categories. The first category consists of approaches that rely on expert knowledge, where researchers manually select potential critical binaries (such as *httpd* and *cgibin*) as objects for security analysis [8-11,15,23-25]. The second category consists of approaches that are based on heuristic methods, automatically identifying critical firmware binaries as objects through feature analysis [12-15]. The third category consists of approaches that dynamically emulate the external communication services [26-29] received in IoT firmware. However, the first category of approaches relies too heavily on expert knowledge, while the third category of approaches requires device emulation, which demands significant computational resources and poses challenges in firmware emulation. This paper primarily focuses on the second category of method, which involves automated identification.

**Comment 4**: An analysis of the time complexity of Algorithm 1 is required.

**Response**: Thanks for your suggestion regarding the time complexity analysis of Algorithm 1. We have revised the manuscript to include the time complexity analysis of Algorithm 1. These changes can be found on Page 12, Lines 451–461.

**Revision text**:

Suppose that binary set *B* contains the number of *b* is *n*, the average number of functions in each binary *b* is *m*, and *k* is the average number of feature extraction operations in each function *f*. The time complexity of Algorithm 1 mainly consists of the following parts: (1) outer loop: traversing each binary b in binary set *B*, with time complexity of $O (n)$; (2) Inner loop: Traversing each function *f* in each binary *b*, with time complexity of $O (m)$; (3) Internal operation: performing feature extraction on each function *f*, including counting the number of basic blocks, branch statements, API calls, and string matches. Assuming the average number of these operations in each function *f* is a constant *k*, the time complexity of the internal operations is $O (k)$. Therefore, the total time complexity of Algorithm 1 is $O (n \times m \times k)$.

**Comment 5**: The relationship between the multimodal features requires further elaboration. Are these features complementary, enhancing the overall detection accuracy, or redundant?

**Response**: We deeply appreciate your precious comment regarding the relationship between the multidimensional features. Upon reflection, we recognize that our initial explanation in the manuscript was not sufficiently clear, which may have led to ambiguity about whether these features are complementary or redundant. To address this, we have revised the manuscript to provide a more detailed discussion of the complementary relationship between multidimensional features and their role in improving detection accuracy. Specifically, we emphasize that multidimensional features complement each other, and through their complementary design, MDFM can comprehensively characterize border binaries. These changes are on Pages 10-11, Lines 374–389.

**Revision text:**

The 15-dimensional features characterize border binaries from different perspectives and exhibit the following complementary relationships:

- Program-level features (*V_0_* and *V_1_*) provide a global perspective, aiding in identifying the binary’s overall behavior and semantics. For example, *V_0_* can preliminarily assess whether the binary may be a border binary by counting the number of APIs related to external input in the function import table.
- Function-level features (*V_2_*~*V_14_*) provide a localized perspective, aiding in identifying specific network parsing functions.
- Feature weight allocation. In the feature of border binaries, function-level features are assigned higher weights, reflecting the importance of network parsing functions in border binaries, while program-level features act as supplementary components to ensure that the model encompasses all possible border binaries.

By utilizing the complementary design of multidimensional features, MDFM accurately and comprehensively characterizes border binary features, enhancing the accuracy and robustness of border binary detection. A detailed list of the feature vectors and their corresponding descriptions is provided in Table 2.

**Reviewer #2**

Reviewer Comments on BBDetector: Intelligent Border Binary Detection in IoT Device Firmware Based on a Multi-Dimensional Feature Model

**Comment 1**: The abstract should provide a concise summary of the research problem, methods, results, and significance. Currently, it lacks clarity on the specific contributions of the study.

**Response**: It’s great that you’ve considered the feedback and added the contributions to the abstract. Clearly outlining the specific contributions of the study is crucial for readers to grasp the research value quickly. We have added the contribution at the end of the abstract as your suggestion.

**Revision text:**

BBDetector provides an accurate method for border binary detection in IoT firmware security analysis, significantly enhancing the pertinence of vulnerability detection, reducing the complexity of firmware security analysis, and providing essential technical support for improving IoT device security.

**Comment 2**: Expand the literature review to include more recent studies on Border Binary detection in IoT devices. Highlight how this proposed research work differentiates from existing research.

**Response**: We sincerely appreciate your valuable comment regarding the literature review. We have expanded our literature review section to include more recent studies on border binary detection in IoT devices and to highlight how our proposed research differentiates from existing research. We believe this will further strengthen the credibility and rigor of our study.

**Revision text**:

Given the extensive length of the revisions, it is challenging to present the complete modifications in the response letter. The specific revised sections can be found on Page 4, Lines 116-122 and Pages 4-5, Lines 160-191.

**Comment 3**: Provide a detailed explanation of the ensemble learning, combining extreme gradient boosting, light gradient boosting machine, and categorical boosting as base learners with random forest as the meta-learner. Include mathematical formulations and pseudocode for better understanding.

**Response**: Thanks for the careful review of the ensemble learning framework in our study. We sincerely apologize for the oversight in not initially providing a detailed explanation of the ensemble learning method, which may have posed challenges for readers in fully understanding the framework. In response to your comments, we have revised the manuscript by adding mathematical formulations for the ensemble learning framework and providing a detailed explanation of the training and prediction phases through the pseudocode in Algorithm 2. These additions aim to enhance the clarity and comprehensibility of our approach, ensuring that readers can better grasp the integration of extreme gradient boosting, light gradient boosting machine, and categorical boosting as base learners, with random forest as the meta-learner.

**Revision text**:

To maintain clarity and conciseness in the response letter, we have not included the complete modifications here due to their extensive length. The specific revised sections are in Pages 14-16, Lines 515-558.

**Comment 4**: Authors should include the hyperparameters of the Gradient boosting model, light gradient boosting machine.

**Response**: We sincerely thank the reviewer for their constructive comments. In the revised manuscript, we have added the parameter settings for XLC-R, including the hyperparameters of the extreme gradient boosting model (XGBoost) and light gradient boosting machine (LightGBM). To obtain the optimal detection model, we employed a grid search technique to determine these hyperparameters. Detailed information about the hyperparameters can be found in Section 6.2 (Experimental setup) on Page 17, Lines 581-585 of the revised manuscript.

**Revision text**:

The configuration adopted by XLC-R was as follows: XGBoost used *100* estimators, with an evaluation metric of *logloss*, a learning rate of *0.1,* and a random state of *42*; LightGBM used *100* estimators, with a learning rate of *0.1*, a maximum depth of *-1* and a random state of *42*; CatBoost used *100* estimators, with a random state of *42*; and RF used *20* estimators, with a maximum depth of *4*, and a random state of *42*.

**Comment 5**: Authors should include the hyperparameters of light gradient boosting machine.

**Response**: Thank you for your advice. We have taken them seriously and added relevant content about the Light Gradient Boosting Machine (LightGBM) in the revised manuscript, particularly providing a detailed explanation of its hyperparameters, including the specific settings for key hyperparameters such as the learning rate and maximum depth. You can find these details in Section 6.2 (Experimental setup). We believe that these additions have made the study more comprehensive and rigorous.

**Revision text**:

The specific details regarding this point are provided in the revision text under Comment 4. To avoid redundancy, we have not repeated them here.

**Comment 6**: Elaborate on the rationale behind the choice of feature selection via MDFM. Explain the feature selection process in detail.

**Response**: We sincerely thank the reviewer for their insightful comments. In the revised manuscript, we have explained the rationale for choosing the multidimensional feature model (MDFM) as the feature selection method. This rationale is based on two key points: (1) network parsing functions as key features: network parsing functions are critical characteristics of border binaries, as they directly reflect the binaries’ interaction behavior with external networks; (2) handling edge cases: some border binaries may not have explicit network parsing functions but instead achieve network parsing functionality through the collaboration of multiple functions. Therefore, MDFM characterizes border binaries through the program-level and the function-level features, ensuring that the model can comprehensively cover all possible border binaries. Additionally, we have further elaborated on the feature selection process in the introduction of the 15-dimensional features (*V_0_~V_14_*).

**Revision text:**

To avoid overwhelming the response letter with extensive revisions, we have provided the complete modifications in the revised manuscript. The rationale behind the choice of feature selection via MDFM is detailed on Page 10, Lines 366-373, while the process of selecting the 15-dimensional features is explained on Pages 11-12, Lines 390-443.

**Comment 7**: The authors should justify why they chose the specific evaluation metrics used in the study. They should also provide a baseline for comparison with other methods.

**Response**: We sincerely thank you for your suggestions regarding using precision, recall, F1 score, ROC, and AUC as evaluation metrics, as well as your recommendation to include baseline methods. We acknowledge that our initial manuscript lacked a clear justification for the choice of evaluation metrics and baseline comparisons, which was an oversight on our part. In response to your comments, we have added an explanation for selecting these metrics in Section 6.5 (Evaluation metrics), and the specific revisions can be found on Pages 18-19, Lines 634-654. Additionally, we have included baseline methods in Section 6.4, with the detailed modifications provided on Page 18, Lines 623-628. These revisions aim to provide a more comprehensive and justified analysis, ensuring that the study’s evaluation framework is robust and comparable to existing methods.

**Revision text**:

**6.5 Evaluation metrics**：

The reason we chose these five metrics is as follows:

*Precision* (Pre) refers to the ratio of the number of border binaries identified correctly to the total number of instances predicted as border binaries. A higher precision indicates that the model is more accurate in its predictions regarding border binaries, resulting in a reduced likelihood of false positives. Reducing false positive rates is crucial in security analysis since incorrect predictions can lead to unnecessary resource consumption. Thus, precision is a key metric for evaluating the effectiveness of border binary detection methods.

*Recall* (R) refers to the proportion of border binaries correctly identified by the model among all border binaries. The higher the recall is, the stronger the performance of the detection model, and the lower the likelihood of false negatives. This is essential for ensuring comprehensive security analysis of border binaries.

*F1 score*(F1) is the weighted average of precision and recall, providing a balanced metric for evaluating the overall performance of a model. Balancing precision and recall is crucial in practical applications, as a model with high precision but low recall may overlook important cases, while a model with high recall but low precision may produce a significant number of false positives.

The ROC curve is the receiver operating characteristic curve. The closer the ROC curve is to the coordinate (0,1), the better the performance of the learner. The AUC value is the area under the ROC curve, and the higher the AUC value, the stronger the model’s ability to distinguish between border and non-border binaries.

**6.4 Baseline**：

We chose Karonte [12] and SaTC [13] as the baseline methods. Karonte represents the first method for identifying border binaries through network parsing functions, whereas SaTC identifies border binaries via front-end string matching. These two methods represent two different technical routes. Furthermore, Karonte and SaTC are commonly used as baselines in border binary detection research [11, 14, 24, 51]. Therefore, we also chose Karonte and SaTC as baseline methods for comparison.

**Comment 8**: Provide a comprehensive description of the datasets used, including their sources, characteristics, and any preprocessing steps taken before model training.

**Response**: We sincerely thank the reviewer for their valuable suggestions regarding the dataset sources, characteristics, and preprocessing steps. We acknowledge that our initial manuscript did not provide a sufficiently clear or comprehensive description of the datasets and preprocessing steps, which may have hindered the reader’s understanding. Regarding the dataset of Section 6.3, we have revised the manuscript to include a more detailed explanation of the dataset sources. Specifically, we employed the firmware collection and unpacking methods described in Section 5.1 to obtain 8,192 file systems and used the border binary collection method outlined in Section 5.2 to gather border binaries. As for the preprocessing steps, we have expanded the description of the preprocessing steps in Section 5.4, detailing the procedures performed before applying the PowerTransformer, such as checking the data types of feature vectors and handling missing values. These revisions aim to provide a clearer and more complete understanding of the datasets and preprocessing pipeline, ensuring transparency and reproducibility in our study.

**Revision text**:

Given the extensive length of the revisions, it is not practical to present the complete modifications in the response letter. The specific sources and characteristics of the dataset have been further elaborated in Section 6.3 of the revised manuscript, as detailed on Page 17, Lines 587-597. Additionally, the preprocessing steps before model training are described in detail on Page 13, Lines 471-474.

**Comment 9**: Authors should describe the experimental setup, including the hardware and software configurations, to ensure reproducibility of the results.

**Response**: We sincerely thank the reviewer for their constructive comments on the experimental setup. We acknowledge that our initial manuscript did not provide a complete description of the software configurations, which was an oversight on our part. Ensuring reproducibility is a critical aspect of our work, and we appreciate your reminder to address this. In response to your comments, we have updated the experimental setup section to include detailed hardware and software configurations. The specific modifications are provided on Page 17, Lines 577-580. These additions aim to provide a comprehensive overview of the experimental environment, enabling others to replicate our results effectively.

**Revision text**:

All experiments were conducted on a server with an Intel® Xeon(R) Silver 4214 CPU @ 2.2.GHz, 256GB RAM, 5.0TB usable HDD, and two NVIDIA V100S GPUs. The server operating system runs on the Ubuntu 22.04 AMD64 operating system, and the runtime environment includes Python 3.8, Binwalk 2.1.1, and Radare2 5.9.1.

**Comment 10**: Incorporating relevant and recent academic sources could strengthen your paper’s validity and give readers more context and background. Some researches listed below which studied similar problems can be discussed: a).GA-mADAM-IIoT: A new lightweight threats detection in the industrial IoT via genetic algorithm with attention mechanism and LSTM on multivariate time series sensor data. b). A novel hybrid autoencoder and modified particle swarm optimization feature selection for intrusion detection in the internet of things network. c). Modified genetic algorithm and fine-tuned long short-term memory network for intrusion detection in the internet of things networks with edge capabilities.

**Response**: We sincerely thank the reviewer for their insightful suggestions on related research. In our initial manuscript, we regrettably overlooked the discussion of object feature characterization and detection model construction, which was an oversight on our part. The reviewer’s recommended articles make me realize the importance of incorporating these aspects to provide a more comprehensive background and strengthen the validity of our work. In response, we have added the existing literature by discussing object feature characterization and detection model construction in the related work section. This includes six additional references, three of which were recommended by the reviewer. These additions have significantly enhanced our paper’s theoretical foundation and provided readers with a more thorough understanding of the research context. The revisions are detailed in Pages 4-5, Lines 160-176.

**Revision text**:

Furthermore, the characterization of object features and the construction of detection models are also essential factors influencing border binary detection. For example, Gemini [31] transforms control flow graphs into numeric feature vectors and uses a graph neural network (GNN) to implement binary vulnerability detection. Li et al. [32] proposed an intrusion behavior detection method based on an ensemble approach, which introduces a leader class and confidence decision ensemble. Saheed et al. [33] combined an autoencoder with modified particle swarm optimization for feature selection and utilized a deep neural network (DNN) for attack detection. Li et al. [34] integrated a composite feature model with graph neural networks to achieve function similarity detection. Saheed et al. [35] [36] employed optimized genetic algorithms and long short-term memory networks for detecting relevant targets. These methods leverage different features and various machine learning models to attain accurate target recognition, providing significant insights for the method proposed in this paper. However, due to the diversity of IoT protocols and the complexity of firmware behavior, existing methods cannot be directly applied to border binaries detection in IoT devices. Therefore, further targeted improvements and optimizations are necessary, considering the unique characteristics of border binaries.

**Comment 11**: A concluding paragraph explaining the research gap is needed in Section 2 related works. A more in-depth comparison highlighting how these works fall short in addressing the challenges the proposed work aims to solve would be beneficial in the end of related work section.

**Response**: Thank you for your valuable reminder to include a summary paragraph in the related work section. We acknowledge that this was indeed an oversight on our part, and we agree that adding a concluding paragraph to highlight the research gap and provide a more in-depth comparison is crucial for framing the contributions of our work. In response to your comments, we have added a revised concluding paragraph at the end of Section 2 (Related work). The specific modifications are detailed on Page 5, Lines 177-191. This addition not only summarizes the limitations of existing works but also clearly outlines how our proposed study addresses these gaps, providing a stronger foundation for understanding the significance and novelty of our research.

**Revision text**:

In summary, existing border binary detection methods have significant room for improvement regarding feature characterization, false negative rates, and intelligence levels. To address the issue of insufficient feature characterization, we designed a multidimensional feature model that incorporates both program-level and function-level features. These features capture the behavior of border binaries from multiple dimensions, significantly enhancing the accuracy and comprehensiveness of feature characterization. To tackle the problem of high false negative rates, we constructed a border binary dataset containing multiple common protocols. By analyzing a large number of border binaries, the dataset’s diversity and representativeness are ensured, thereby improving the detection capability of the model in different protocol scenarios. To enhance the intelligence level, we adopted a stacking method based on ensemble learning, which combines XGBoost, LightGBM, and CatBoost as base learners, with RF as the meta-learner. By stacking multiple machine learning models for border binary prediction, BBDetector can more effectively learn the key feature attributes of border binaries, further enhancing detection accuracy and robustness.

**Comment 12**: Please Change the “conclusion” section to “Conclusion and Future Work” and write future work.

**Response**: We sincerely thank the reviewer for their constructive suggestion to extend the conclusion section with a discussion of future work. The revised ‘Conclusion and future work’ section is provided on Pages 24-25, Lines 823-841, with the subsection on future work starting on Page 25, Line 835.

**Revision text**:

In future studies, we aim to optimize existing methods. First, we plan to expand the MDFM by incorporating additional important features while examining the impact of the current 15-dimensional features on false negatives and eliminating feature dimensions that cause high interference with the detection results. Second, we intend to collect more border binaries through the application of BBDetector to enrich our sample set and continually optimize the existing detection models via iterative training methods to improve the detection accuracy of border binaries.

**Comment 13**: Numerical results are good enough, but more explanations are required to analyze each figure presented.

**Response**: We sincerely thank the reviewer for their valuable suggestion to provide a more detailed interpretation of the numerical results and figures. Based on this comment, we have conducted a comprehensive and meticulous review and revision of the analysis of numerical results for the four discussions of experiments in Section 6.6. By supplementing relevant details, we aim to present the research findings in a clearer and more accessible manner to readers.

**Revision text**:

Since the newly added content is distributed across multiple subsections, we have highlighted these additions in yellow in the revised manuscript to facilitate your review. The highlighted sections begin on Page 19, Line 656.

**Comment 14**: Proofread the manuscript carefully to eliminate any grammatical errors or typos and ensure clarity and coherence in writing. Additionally, adhere to the formatting and style guidelines specified by the journal to enhance the professionalism of the manuscript.

**Response**: Thank you for your valuable suggestions. After completing the revisions for all the comments, we attached great importance to improving the quality of the manuscript and conducted meticulous proofreading of the entire draft. During the proofreading process, we identified and corrected several grammatical errors. Meanwhile, we reorganized and polished some sentences that were not clear or coherent enough to ensure a smooth, logical flow and explicit expression of ideas in the article. In addition, we thoroughly checked and adjusted the manuscript in strict accordance with the formatting and style guidelines specified by the journal, making every effort to enhance its professionalism. After these careful revisions, the manuscript has seen further improvements in both language quality and formatting standards.

Once again, we sincerely thank the reviewers for their valuable comments, which have significantly improved the quality of our manuscript. We hope the revised version meets the journal’s standards and look forward to your feedback.

Sincerely,

Guimin Zhang, PhD

School of Cybersecurity

Information Engineering University, China
