# Supplementary material for: BBDetector: Intelligent border binary detection in IoT device firmware based on a multidimensional feature model
Source: PLoS One. 2025 Aug 7;20(8):e0329469. doi: 10.1371/journal.pone.0329469 (PMC12331098; doi:10.1371/journal.pone.0329469)
Supplement: Attachment — Submitted filename: Response_to_Reviewers_auresp_2.docx [file pone.0329469.s002.docx]

Response to Academic Editor and Reviewers

July 9, 2025

Guimin Zhang

Email: zh.guimin@163.com

Manuscript Number: [PONS-D-24-50757R1]

BBDetector: Intelligent border binary detection in IoT device firmware based on a multidimensional feature model

Dear Editors and Reviewers,

Thank you for your time and effort in reviewing our work and constructive suggestions on our manuscript. Based on your comments, we have carefully revised the manuscript to address all the issues raised. All revisions have been highlighted in yellow in a separate file labeled “Revised Manuscript with Track Changes”. Below, we provide a point-by-point response to your comments and describe the changes made to the manuscript.

**Academic Editor**

You are requested to revise your manuscript as per the comments.

**Comment 1**: **Insufficient Contextualization: The paper would benefit from a stronger connection to the existing literature. The introduction and related work section should explicitly discuss how BBDetector builds upon, differs from, or improves upon recent advances in IoT firmware security and border binary detection.**

**Response**: Thank you very much for your valuable feedback. You suggest that we clarify in the introduction and related work sections how BBDetector is built, as well as how it differs from and improves upon recent advances, which is highly valuable. To ensure readers better understand BBDetector’s contribution, it is important to clearly situate it within the current research on IoT firmware security and border binary detection. In the revised manuscript, we have made the following adjustments based on your suggestions:

In the introduction section, we first updated the data cited on the number of IoT devices in the research background, replacing the data originally based on 2024 statistics with the latest forecast value for 2025 to more accurately reflect the current situation. Second, when explaining that Linux-based firmware images are essential for IoT devices, we replaced the cited literature. The original citation of Costin et al.'s research results published in 2014 was changed to a citation of Gibbs et al.'s research results published at USENIX Security 2024. Both articles emphasize the importance of Linux-based firmware, but Mango represents the latest progress in firmware analysis methods, which better reflects the foundation of current research. More importantly, we added detection methods for IoT firmware developed in recent years (such as SinkTaint 2023, SLFHunter 2024, and LATTE 2025), highlighting the necessity of researching border binary detection methods.

In the related work section, we first removed some outdated but thematically similar references, such as DTaint 2018, Saluki 2018, to ensure the timeliness of the relevant content. Then, we expanded the literature coverage in the field of IoT firmware analysis by including significant recent research methods, such as SinkTaint 2023, SLFHunter 2024, and LATTE 2025. Lastly, we supplemented the research methods that are enlightening for border binary detection, as recommended by the editor. These additions aim to establish a more solid theoretical foundation for this study and indicate directions for further exploration.

We believe that the background introduction of the paper is more comprehensive after these revisions, the connection between BBDetector and existing research is clearer, and its innovation and value are better reflected.

**Comment 2**: **Limited Recent References: The current reference list appears to lack sufficient representation of recent publications (2023-2025) in the specific area of IoT firmware analysis and vulnerability detection. This limits the paper's ability to demonstrate its novelty and relevance.**

**Response**: Thank you very much for your valuable feedback on reference timeliness. We fully understand that citing the latest research methods is crucial for clearly demonstrating the novelty and relevance of this paper to the current research frontier. You mentioned that our reference list does not sufficiently represent the field of IoT firmware analysis and vulnerability detection from 2023 to 2025, which is an area that needs improvement.

Based on your suggestion, we have retrieved and supplemented relevant research methods published in the field of IoT firmware analysis in recent years, especially from 2023 to 2025. We have also replaced some relatively outdated references with more representative and recent ones. While incorporating these new references, we also adjusted the discussion in the introduction and related work sections accordingly to better reflect the current research landscape. Additionally, we highlighted the unique contributions and advantages of BBDetector through analysis and comparison with these updated references. Through these modifications, we believe that the references now better capture the latest developments in the field and demonstrate the novelty and contribution of our method.

Deleted literature includes:

(1) Costin A, Zaddach J, Francillon A, Balzarotti D. A Large-Scale Analysis of the Security of Embedded Firmwares. USENIX Association; 2014. p. 95–110.

(2) Cheng K, Li Q, Wang L, Chen Q, Zheng Y, Sun L, et al. DTaint: Detecting the 873 Taint-Style Vulnerability in Embedded Device Firmware. IEEE Computer Society; 2018. p. 430–441.

(3) Gotovchits I, Van Tonder R, Brumley D. Saluki: Finding Taint-style 876 Vulnerabilities with Static Property Checking. In Proceedings of the NDSS Workshop on Binary Analysis Research; 2018.

Add literature excluding the references listed in Comment 3:

(1) Gibbs W, Raj AS, Vadayath JM, Tay HJ, Miller J, Ajayan A, et al. Operation Mango: Scalable Discovery of Taint-Style Vulnerabilities in Binary Firmware Services. USENIX Association; 2024.

(2) Yin X, Cai R, Zhu X, Yang Q, Song E, Liu S. Precise Discovery of More Taint-Style Vulnerabilities in Embedded Firmware. IEEE Trans Dependable Secur Comput. 2025;22(2):1365–1382.

(3) Liu P, Sun C, Zheng Y, Feng X, Qin C, Wang Y, et al. LLM-Powered Static Binary Taint Analysis. ACM Trans Softw Eng Methodol. 2025;34(3):83:1–83:36.

(4) Qasem A, Debbabi M, Soeanu A. OctopusTaint: Advanced Data Flow Analysis for Detecting Taint-Based Vulnerabilities in IoT/IIoT Firmware. ACM; 2024. p. 2355–2369.

(5) Ye J, Fei X, de Carn´e de Carnavalet X, Zhao L, Wu L, Zhang M. Detecting command injection vulnerabilities in Linux-based embedded firmware with LLM-based taint analysis of library functions. Comput Secur. 2024;144:103971.

**Comment 3**: **Suggested Citations: The authors should consider incorporating the following recent** **articles and others identified through a more comprehensive literature search.**

1. Xia, J., Li, S., Huang, J., Yang, Z., Jaimoukha, I. M.,... Gündüz, D. (2023). Metalearning-Based Alternating Minimization Algorithm for Nonconvex Optimization. IEEE Transactions on Neural Networks and Learning Systems, 34(9), 5366-5380. doi: 10.1109/TNNLS.2022.3165627

2. Zhou, W., Xia, C., Wang, T., Liang, X., Lin, W., Li, X.,... Zhang, S. (2025). HIDIM: A novel framework of network intrusion detection for hierarchical dependency and class imbalance. Computers & Security, 148, 104155. doi: https://doi.org/10.1016/j.cose.2024.104155

3. Zhou, Z., Li, Z., Zhou, W., Chi, N., Zhang, J.,... Dai, Q. (2025). Resource-Saving and High-Robustness Image Sensing Based on Binary Optical Computing. Laser & Photonics Reviews, 19(7), 2400936. doi: https://doi.org/10.1002/lpor.202400936

4. Huang, S., Sun, C., & Pompili, D. (2025). Meta-ETI: Meta-Reinforcement Learning with Explicit Task Inference for UAV-IoT Coverage. IEEE Internet of Things Journal. doi: 10.1109/JIOT.2025.3553808

5. Zhang, Z., Liu, Z., Martin, A., & Zhou, K. (2023). BSC: Belief Shift Clustering. IEEE Transactions on Systems, Man, and Cybernetics: Systems, 53(3), 1748-1760. doi: 10.1109/TSMC.2022.3205365

6. Liu, Y., Huo, M., Li, M., He, L., & Qi, N. (2025). Establishing a Digital Twin Diagnostic Model Based on Cross-Device Transfer Learning. IEEE Transactions on Instrumentation and Measurement, 74, 1-10. doi: 10.1109/TIM.2025.3562973

7. Zhang, H., Xu, Y., Luo, R., & Mao, Y. (2023). Fast GNSS acquisition algorithm based on SFFT with high noise immunity. China Communications, 20(5), 70-83. doi: 10.23919/JCC.2023.00.006

8. Qiao, Y., Lü, J., Wang, T., Liu, K., Zhang, B.,... Snoussi, H. (2024). A Multihead Attention Self-Supervised Representation Model for Industrial Sensors Anomaly Detection. IEEE Transactions on Industrial Informatics, 20(2), 2190-2199. doi: 10.1109/TII.2023.3280337

9. Zhang, M., Wei, E., Berry, R., & Huang, J. (2024). Age-Dependent Differential Privacy. IEEE Transactions on Information Theory, 70(2), 1300-1319. doi: 10.1109/TIT.2023.3340147

10. Li, X., Lu, Z., Yuan, M., Liu, W., Wang, F., Yu, Y.,... Liu, P. (2024). Tradeoff of Code Estimation Error Rate and Terminal Gain in SCER Attack. IEEE Transactions on Instrumentation and Measurement, 73, 1-12. doi: 10.1109/TIM.2024.3406807

11. Jiang, N., Feng, Q., Yang, X., He, J., & Li, B. (2025). The octonion linear canonical transform: Properties and applications. Chaos, Solitons & Fractals, 192, 116039. doi: https://doi.org/10.1016/j.chaos.2025.116039

**Response**: Thank you very much for providing the list of suggested citations. We have carefully reviewed each article you recommended and found that these articles indeed represent recent advances in the relevant field, offering us valuable perspectives for examining existing work. After thorough evaluation, we included the literature you recommended in the revised manuscript and added several other relevant literature to make the related work section more comprehensive and to provide stronger theoretical support for the proposed method.

In the related work section, we explored the impact of two key factors, object feature characterization and detection model selection, on the performance of border binary detection. To do this, we added the second recommended paper, a network intrusion detection framework based on hierarchical dependency and class imbalance, after object feature characterization, to gain insights into its approach to handling feature relationships. Simultaneously, we included the first recommended paper on meta-learning, the third on photonic neural networks, the fourth on meta-reinforcement learning, the fifth on belief transfer clustering, the sixth on transfer learning, and the eighth on multi-head attention self-supervised representation models into the section on detection model construction. By comparing the advantages of these state-of-the-art models, the rationality of the selected detection model in terms of adaptability will be more comprehensively demonstrated. In addition, we proactively supplemented several important relevant studies on IoT firmware analysis published over the past three years.

In the conclusion and future work section: To explore diverse technological paths for enhancing the accuracy of border binary detection, we taken the seventh and eleventh recommended papers on signal acquisition and processing methods, the tenth recommended paper on attack detection, and the ninth recommended paper on differential privacy as key references for future work. The goal is to provide potential research directions for improving the accuracy of border binary detection.

By supplementing and integrating literature, the reference list can better showcase the latest advancements in firmware security and related areas. This not only improves the timeliness and academic rigor of the paper but also more clearly highlights the unique contribution and research value of BBDetector compared to directly related work in this field.

Thank you again for providing valuable suggestions. These references have greatly inspired our understanding and thinking regarding border binary detection methods.

**Reviewer #1**

**The authors have addressed all my comments, and this paper is improved significantly, so I think this paper can be accepted for publication in this journal.**

**Response**: We sincerely appreciate your recognition and recommendation. It is truly thanks to the valuable advice you offered earlier that we feel more confident in presenting this revised manuscript to the readers.

**Reviewer #2**

**The authors have addressed all the comments. The authors have addressed all the comments. The authors have addressed all the comments. The authors have addressed all the comments. The authors have addressed all the comments. The authors have addressed all the comments. The authors have addressed all the comments. The authors have addressed all the comments. The authors have addressed all the comments.**

**Response**: Thank you to the reviewer for recognizing our work. As you mentioned, we have addressed and responded to every comment received. We appreciate every reviewer’s opinion and have made every effort to ensure that all feedback has been carefully considered.

**Reviewer #3**

**Authors did well to address comments. They addressed all comments highlighted previously and gave proper response to each alongwith changes made.**

**Response**: We thank the reviewer for their thorough observations and helpful suggestions regarding our response. It is true that we put significant effort into revising the manuscript to improve its quality and clarity. We appreciate your encouraging words and valuable professional insights.

Once again, we sincerely thank the editors and reviewers for their valuable comments, which have significantly improved the quality of our manuscript. We hope the revised version meets the journal’s standards and look forward to your feedback.

Sincerely,

Guimin Zhang, PhD

School of Cybersecurity

Information Engineering University, China
